# BRF Negatively Regulates Thermotolerance Defect of *fes1a* in *Arabidopsis*

**DOI:** 10.3389/fpls.2020.00171

**Published:** 2020-03-10

**Authors:** Can Fu, Xiaxia Liu, Xuezhi Li, Panfei Huo, Jingjing Ge, Yanfei Hou, Wenwen Yang, Jingxia Zhang, Limin Zhang, Dazhong Zhao, Changle Ma, Jian Liu

**Affiliations:** ^1^ College of Life Sciences, Shandong Normal University, Jinan, China; ^2^ Cotton Research Center, Shandong Academy of Agricultural Sciences, Jinan, China; ^3^ Department of Biological Sciences, University of Wisconsin-Milwaukee, Milwaukee, WI, United States

**Keywords:** *Arabidopsis thaliana*, BRF, FES1A, RNA Polymerase II, RNA Polymerase III, heat stress

## Abstract

FES1A is a heat shock protein 70 binding protein. Mutation of *FES1A* leads to a defect in thermotolerance of *Arabidopsis*; however, independent *fes1a* mutants exhibit a range in the extent of thermosensitivity. Here, we found that *BRF2*, a gene adjacent to *FES1A* and encoding a component of transcription factor IIIB, affects the thermosensitivity of *fes1a* mutants. Knockout of *BRF2* suppressed *fes1a* thermosensitivity, while overexpression of *BRF2* increased thermosensitivity of *fes1a.* BRF2 in *fes1a* mutants regulates the transcriptional strength of RNA Polymerase II and accumulation of heat shock proteins and eventually affects the thermotolerance of *fes1a*. There is a cross-talking between RNA Pol III and Pol II. The cross-talking is initiated by *BRF*, magnified by the mutation of *FES1A,* and finally has an effect on thermotolerance.

## Introduction

Plants are sessile organisms. To cope with high-temperature stress, plants have evolved various adaptive mechanisms to optimize their growth and development to ultimately achieve successful reproduction. Thermotolerance in plants is associated with a number of regulatory and functional genes which constitute complex signaling pathways. It has been found that abscisic acid, salicylic acid, ethylene, reactive oxygen species, membrane fatty acid composition, ubiquitin, heat shock proteins (HSPs), programmed cell death, and photooxidation are all involved in thermotolerance.

Out of numerous heat response-related factors, HSPs are the best understood. HSPs function as molecular chaperones to prevent cellular proteins from denaturation and to promote the refolding of damaged proteins. During heat stress, accumulation of HSPs confers plant cells with acquired thermotolerance, which enables plants to survive under severe heat stress. While acquiring thermotolerance, different classes of HSPs function in different ways. For example, HSP101 is mainly responsible for dispersing the aggregates of heat-denatured proteins (Merret et al., 2017). Cytosolic HSP90 regulates the heat shock response (Mclellan et al., 2007). Once HSP90 chaperon complex becomes inactivated, the *Arabidopsis* mutant shows a defect in thermotolerance (Fernández-Bautista et al., 2018). HSP70 is a multifunctional factor. Apart from preventing the aggregation of denatured proteins and assisting the refolding of heat-denatured proteins (Saibil, 2013; Mayer and Gierasch, 2019), HSP70 interacts with HSFs, cochaperones, and other proteins to coordinate complex signaling networks (Zuiderweg et al., 2017). Disruption of HSP70 function decreases plant thermotolerance (Su and Li, 2008), while overexpression of HSP70 confers multiple abiotic stress tolerance in transgenic *Arabidopsis thaliana* (Masand and Yadav, 2016).

HSP70 contains a highly conserved N-terminal nucleotide-binding domain (NBD) and a substrate-binding domain (SBD). ATPase activity is contained in the NBD. In the ATP-bound state, the SBD of HSP70 loosely associates with its substrates (Gowda et al., 2018). Once ATP is hydrolyzed into ADP, the SBD changes conformation and tightly binds to its substrate. When ADP is released from the NBD, HSP70 liberates its substrate and folds back into the natural state to start its next catalytic cycle. In the cycle of HSP70 function, the disassociation of ADP from HSP70 is a rate-limiting step that is accelerated by nucleotide exchange factors (NEFs) (Dragovic et al., 2006). FES1 is a Cytosolic NEF. In addition, FES1 is involved in degradation of misfolded cytosolic proteins (Gowda et al., 2013; Gowda et al., 2016). *Arabidopsis* FES1A is a HSP70 binding protein and is able to complement the heat-sensitive defect of the *Saccharomyces cerevisiae fes1Δ* strain. In *Arabidopsis*, knockout of *FES1A* results in an increase in heat-stress susceptibility (Zhang et al., 2010).

Eukaryotic genes and RNAs are transcribed by three RNA polymerases. RNA polymerase (Pol) I and Pol II are responsible for transcribing rRNAs and mRNAs, respectively, while RNA Pol III transcribes tRNAs, 5S ribosomal RNAs, 7SL RNAs, RNase P, and U6 spliceosomal small nuclear RNAs (Dieci et al., 2013; Duttke, 2014). Genes transcribed by RNA Pol III are involved in ribosome biogenesis, RNA processing, and chromatin regulation (Willis and Moir, 2018). RNA Pol III is a large complex (Hoffmann et al., 2016), in which transcription factor IIIB (TFIIIB) is a central transcription initiation unit. TFIIIB is composed of three proteins: TATA-binding protein (TBP), TFIIIB-related factor (BRF), and B-double prime 1 (BDP1) (Khoo et al., 2014). Yeast BRF1 is involved in thermotolerance (Conesa et al., 2005). Redox sensing by BRF2 modulates resistance to oxidative stress in normal and cancer cells (Gouge et al., 2015). However, no research on plant BRF in stress tolerance was presented.

In our previous studies, two T-DNA insertion mutants of *fes1a* exhibited defects in thermotolerance but to different degrees (Zhang et al., 2010). In this study, three independent *fes1a* T-DNA insertion mutants were used to evaluate the mechanisms of thermotolerance. Interestingly, three *fes1a* mutants exhibited different extents of thermo-sensitivity. Using allelic hybridization and evaluation of double mutants, we found that the factor suppressing the thermo-sensitivity of *fes1a* mutants was *BRF2*, a gene adjacent to *FES1A*, which encodes a component of TFIIIB. Further analysis showed that the amount of BRF2 in the absence of FES1A affected the activity of RNA Pol II, which ultimately regulates the response of thermotolerance-related molecules, such as HSPs, and modulates the thermotolerance in *fes1a*. These results provide important evidence that BRF of the Pol III in plant regulates thermotolerance.

## Materials and Methods

### Plant Material and Growth Conditions

*Arabidopsis thaliana* mutants (**Supplemental Table 1**) were obtained from the ABRC (http://abrc.osu.edu). T-DNA insertions were confirmed using PCR and sequencing (**Supplemental Table 2**). After backcrossing twice with WT, the mutants were used for the experiments. The thermotolerance of seedlings was evaluated according to the intermittent temperature-increasing regime. First, ten-day-old plants were acclimated at 38°C for 2 h and then subjected to lethal heat treatment at 45°C for different time periods. Following that, the plants were transported to a growth chamber under normal growth conditions for recovery. The phenotypes were photographed after seven days of recovery (Zhang et al., 2010).

### Yeast Complementation Assay

The recombinant yeast strains and plasmids used in this study are listed in **Supplemental Table 3**. The primers used are shown in the **Supplemental Table 4**. The *ScBRF1-ΔN10* cDNA fragment (lacking the coding sequence for the first 10 amino acids) was cloned into pYX242WS (Gibson, 2011; Wang et al., 2013), generating the plasmid *ScBRF1-ΔN10*-pYX. The *Arabidopsis BRF2* cDNA sequence was ligated into plasmid pJFE3 (Shen et al., 2012) to generate the plasmid *AtBRF2*-pJFE3. The deletion of *ScBRF1* was generated *via* the Cre-loxP method (Hegemann et al., 2006).

### Generation of *Arabidopsis* Transgenic Plants

To generate the *BRF2* overexpression construct, the open reading frame of *BRF2* was amplified by PCR (**Supplemental Table 5**) and then inserted into the *Afl* II site of plasmid pMY72, resulting in the plasmid *BRF2-(HA)×2*-pMY72. The PCR-amplified fusion fragment of *BRF2-(HA)×2* (**Supplemental Table 5**) was inserted into the *Eco*R I and *Kpn* I sites of *P_LeHsp23.8_*-pRT101 (Yi et al., 2006), generating the plasmid *P_LeHsp23.8_*-*BRF2-HA*_2_-pRT101. *Arabidopsis* was transformed using the floral dipping method (Clough and Bent, 1998).

The *BRF2* gene in the *fes1a* background was mutated using CRISPR/Cas9 (Xing et al., 2014). The *BRF2*-CRISPR/Cas9 cassette was generated by PCR amplification from pCBC-D1T1. The PCR products were digested with *Bsa* I and ligated into pHSE401, generating the plasmid *BRF2*-pHSE401 that was used for *Arabidopsis* transformation. The transgenic lines were selected using hygromycin B and further confirmed by enzyme digestion and sequencing. All primers used are listed in **Supplemental Table 5**.

### Subcellular Localization of BRF2

The cDNA of *BRF2* was amplified by PCR (**Supplemental Table 5**) and then inserted into the *P_35sCaMV_*-*GFP* vector, generating the fusion construct *P_35sCaMV_-BRF2*-*GFP*. The fusion construct was transformed into *Arabidopsis* by the floral dipping method. The subcellular localization of the BRF2-GFP fusion was examined by confocal laser scanning microscopy (Leica TCS SP8).

### RNA Extraction, RT-PCR, and qRT-PCR

Total RNA was isolated from 15-day-old seedlings using TRIzol reagent (Invitrogen). First-strand cDNA was synthesized using a PrimeScript™ RT Reagent Kit with a gDNA Eraser (Takara). RT-PCR was performed using the primers shown in **Supplemental Table 6**. qRT-PCR was performed on a Roche LightCycler^®^ 480 (LC480) using the SYBR Green system. The gene specific primers used in the qRT-PCR analysis are listed in **Supplemental Table 7**. The *ACTIN8* gene was used as an internal control.

### Immunoblotting Analysis

Western blot was performed as described previously (Zhang et al., 2010) using the primary antibodies anti-FES1A antibody, anti-HSC70 monoclonal antibody (SPA-817; Stressgen, San Diego, CA, US), anti-HSP101 N-terminal (AS07 253; Agrisera, Vännäs, Sweden), anti-CLPB N-terminal (Yang et al., 2006), anti-small HSP class II antibody (AS07 255; Agrisera), anti-HSP21 antibody (AS08 285; Agrisera), anti-ACTIN antibody (AB10007; Sangon, Shanghai, China), anti-HISTONE 3 antibody (AS10 710; Agrisera), anti-HA antibody (Ab9110; Abcam), anti-S2P-CTD (ab5095, Abcam), and anti-S5P-CTD (ab5131, Abcam) antibodies. Anti-BRF antibody (rabbit) was generated using the C-terminal fragment of BRF2 (361-604 amino acids).

### Statistical Analysis

All experiments in this study were performed at least three times, and the values are expressed as the mean ± SD. Statistical significance was determined by Duncan's multiple range test (*P* < 0.05).

## Results

### The *fes1a* Mutants Exhibit Differential Sensitivities to Heat Stress

**Figure 1:**
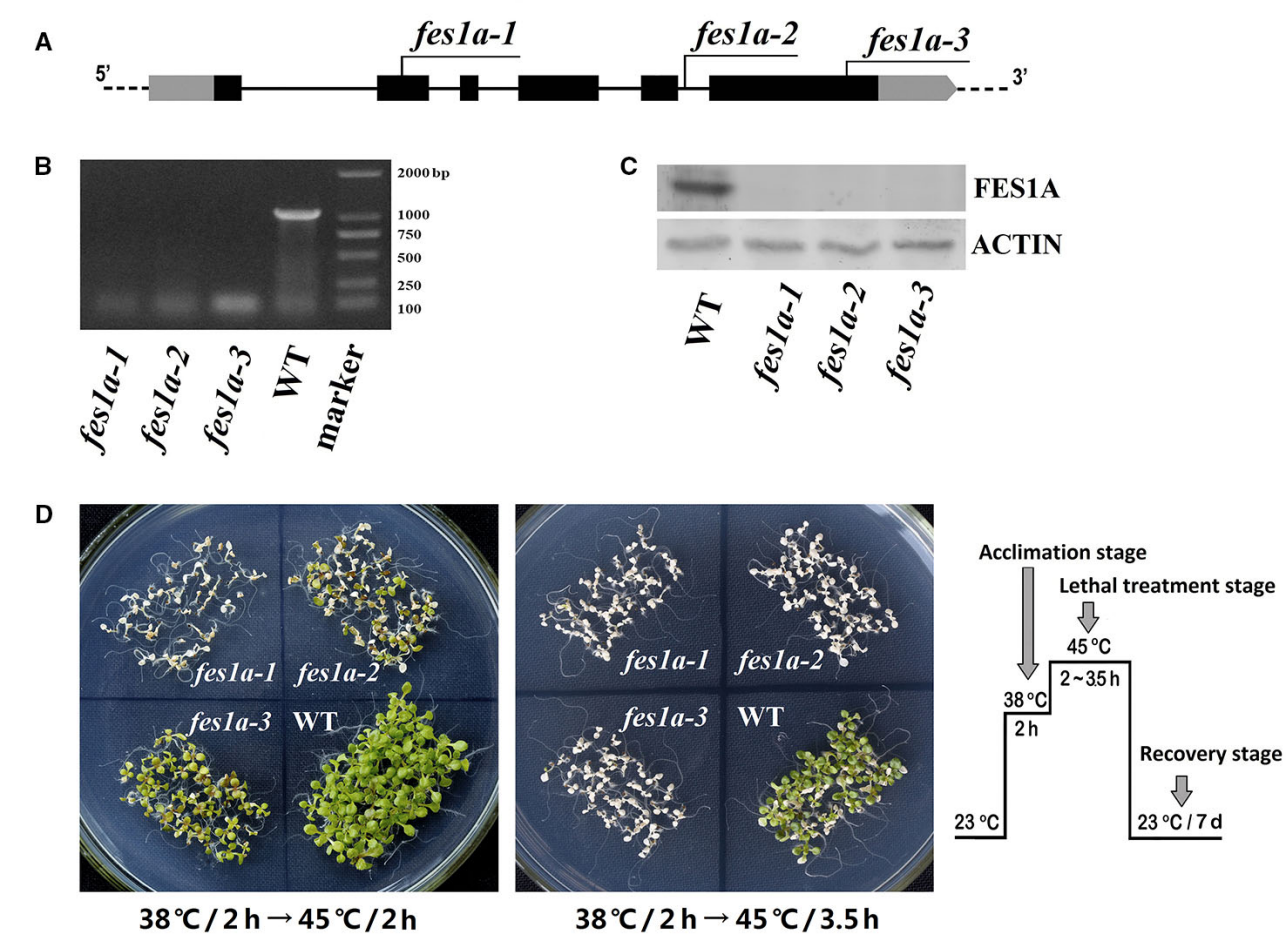
Differences in thermotolerance of the three *fes1a* mutants. **(A)** A diagram showing T-DNA insertions in three examined *fes1a* mutants. Exons are black boxes, untranslated coding regions are grey boxes, and introns are lines. RT-PCR **(B)** and western blots **(C)** exhibiting transcription and translation of *FES1A*, respectively. Plants were treated at 38 °C for 2 h before total RNA and protein extraction. **(D)** Evaluation the thermotolerance of *fes1a* mutants. A temperature-increasing regime for thermotolerance evaluation is schematically shown on the right. Ten-day-old plants were first acclimated at 38 °C for 2 h and then subjected to lethal heat treatment at 45 °C for 2~3.5 h as indicated in the center of each petri dish. After heat stress, plants were transferred to a growth chamber under normal growth conditions for recovery and were photographed after seven days of recovery.

In our previous study, we examined the thermotolerance of two allelic mutants of *fes1a*, each of which contained a T-DNA insertion in the sixth exon of *FES1A* (Zhang et al., 2010). Both mutants showed defects in thermotolerance, but to significantly different degrees. To investigate the cause of variability in thermotolerance of the *fes1a* mutants, we chose three allelic mutants for further thermotolerance evaluation (**Figure 1**). Sequencing results revealed that *cs842189* had a T-DNA insertion in the second exon, while *salk_012416* in the fifth intron and *salk_072075* harbored a T-DNA insertion in the sixth exon (**Figure 1A**; **Supplemental Figure 1**). To distinguish these three *fes1a* mutants, we sequentially renamed them *fes1a-1* to *fes1a-3* based on the T-DNA locations from 5′- to 3′-UTR (**Figure 1A**, **Supplemental Table 1**).

RT-PCR and western blot showed that the *FES1A* transcript and FES1A protein were absent from all three *fes1a* mutants (**Figures 1B, C**), suggesting that these *fes1a* mutants are loss-of-function mutants. T-DNA insertions in different sites did not result in the formation of any truncated FES1A proteins, since no small FES1A fragments was detected in any of the *fes1a* mutants using the anti-FES1A antibody (**Supplemental Figure 2**), which recognizes both the N-terminal and the C-terminal of FES1A (Zhang et al., 2010).

We evaluated the heat sensitivities of all three *fes1a* mutants. Ten-day-old seedlings were first subjected to heat acclimation at 38°C for 2 h, and then exposed to lethal heat-stress at 45°C for different lengths of time. Upon 2 h of lethal stress at 45°C, *fes1a-1* and *fes1a-2* both exhibited symptoms of severe damage (**Figure 1D**), while *fes1a-3* displayed great resistance to heat stress. When the lethal heat-stress was extended to 3.5 h, all three *fes1a* mutants, but not the wild-type (WT) plants, finally perished, indicating that all *fes1a* mutants possessed thermotolerance defects, but with different sensitivities. In addition, the survival rates of the seedlings undergoing an acquired thermotolerance challenge indicated that *fes1a-3* had the best thermotolerance among the three *fes1a* mutants, with the survival rate reaching 92.5%, while *fes1a-2* and *fes1a-1* had 41.7% and 0.8% survival, respectively (**Supplemental Figure 3A**). These phenotypes (**Figure 1D**) and physiological data (**Supplemental Figure 3**) confirmed our previous results that the null mutants of *FES1A* exhibited differential extents of thermotolerance (Zhang et al., 2010).

### The Differential Thermotolerance of *fes1a* Mutants Is Closely Linked With Their Adjacent *BRF2* Gene

Although *fes1a-3* phenotype exhibited its significantly higher thermotolerance than those of *fes1a-1* and *fes1a-2,* we clearly observed the thermotolerant difference between *fes1a-1* and *fes1a-2*. In other words, *fes1a-2* was more tolerant than *fes1a-1* (**Figure 1D**, **Supplemental Figure 3A**). Accordingly, the factor modulating the thermotolerance should be ubiquitous in all the three *fes1a* mutants. A simple explanation for the observation was that the involved factor might genetically link to *FES1A* locus. Thus, we performed two sets of allelic hybridization experiments, in which three independent lines of *fes1a* were included. After selecting out homozygous F2 progenies, more than 100 homozygous F2 progenies were evaluated for thermo-sensitivity. As a result, the thermotolerance of F2 progenies was exactly determined by the genotype of the T-DNA insertion. In other words, if the genotype of the T-DNA insertion in an F2 individual was the same as its parent line, the parent and the offspring showed identical thermotolerance (**Supplemental Table 8**). Therefore, this suggests that the factor differentiating the thermo-sensitivities of the *fes1a* mutants was closely linked to the *FES1A* locus or to the individual location of T-DNAs. As each of the T-DNAs in *fes1a* could exert a significant effect on the thermo-sensitivities of the *fes1a* mutants, the genes possibly influenced by the T-DNA insertions should be confined to the outer edge of *FES1A*. Using qRT-PCR, we examined the expression of the proximal genes adjacent to *FES1A* in the *fes1a* mutants. Only *BRF2* (Lagrange et al., 2003), which encodes one of the three components of transcription factor IIIB, was significantly downregulated in comparison to WT (**Supplemental Figure 4**).

**Figure 2:**
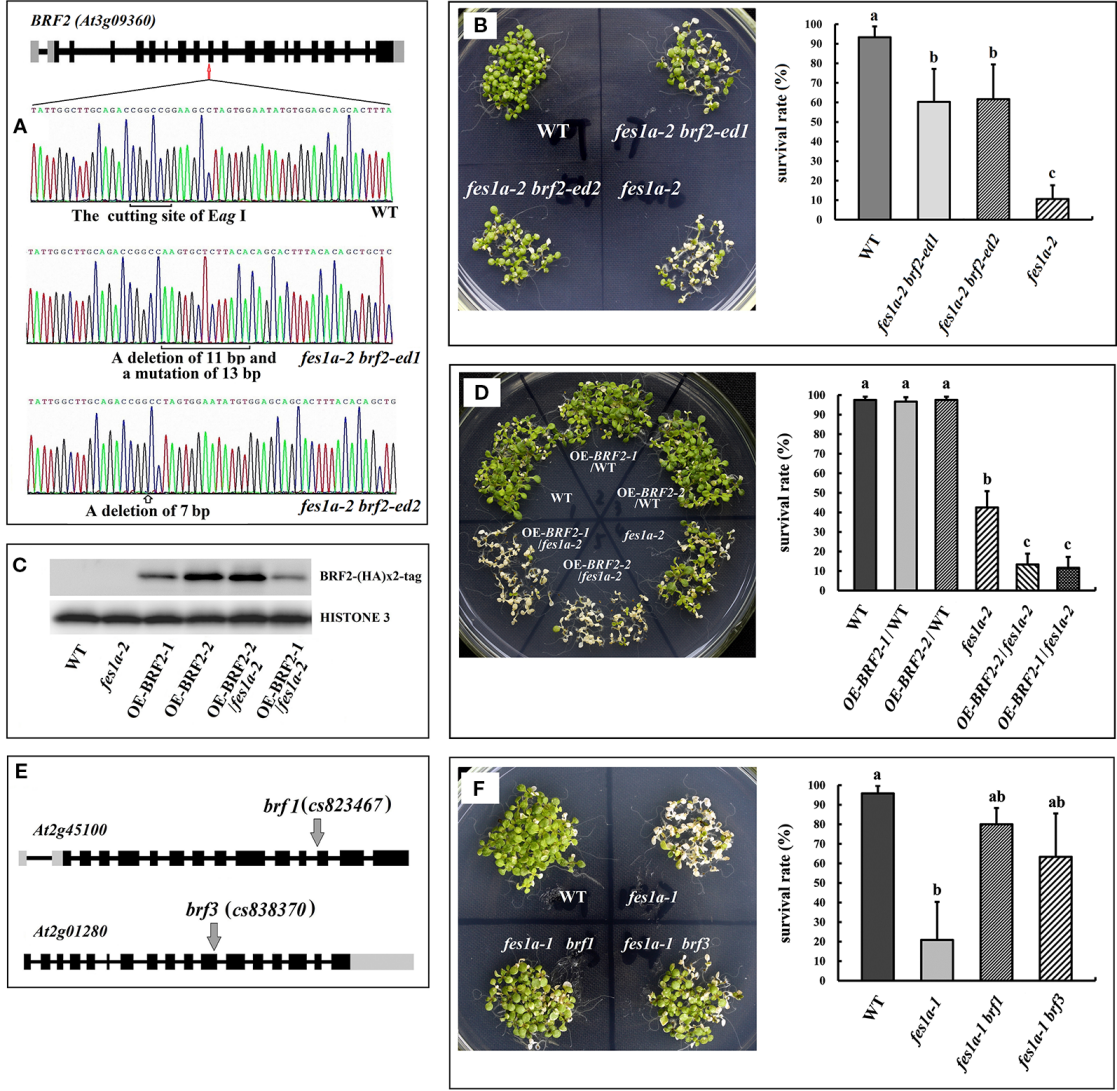
*BRF* genes suppress the thermosensitivity of *fes1a* mutants. **(A)** Sequencing results revealed CRISPR/Cas9 generated mutations (at 10^th^ exon) in the *BRF2* gene in *fes1a-2 brf2-ed1* and *fes1a-2 brf2-ed2* double mutants. **(B)** The *fes1a-2 brf2-ed1* and *fes1a-2 brf2-ed2* mutants showing enhanced thermotolerance in comparison with the *fes1a-2* mutant (left). The corresponding survival rate is on the right. **(C)** Western blot result displaying expression of the BRF2-(HA)×2 protein in overexpression transgenic lines. HISTONE 3 was used as a loading control. **(D)** Overexpression of *BRF2* decreased the thermotolerance of the *fes1a-2* mutant, but had no effect on wild-type (left). The corresponding survival rate is on the right. **(E)** A diagram illustrating T-DNA insertions in *BRF1* and *BRF3* gene. **(F)** Mutation in *BRF1*and *BRF3* genes improved thermotolerance in the *fes1a-1* mutant (left). The corresponding survival rate is on the right. Data in **(B, D, F)** represent the means of 3 replicates ± SD. For each column, different letters *a*, *b*, *c* indicate significant differences at *P* < 0.05. The temperature regime of heat stress in **(B, D, F)** was the same as shown in **Figure 1**, except that the lethal treatment at 45°C lasted for 2 h in **(B)**, 1.5 h in **(D)**, and 2 h in **(F)**.

In order to investigate whether *BRF2* is responsible for regulating thermotolerance in *fes1a* mutants, we knocked out the *BRF2* gene using the CRISPR/Cas9 system in the *fes1a-2* mutant background (**Figure 2**). DNA sequencing revealed that the *fes1a-2 brf2-ed1* double mutant had 11 base pairs deletion and 13 base pairs mutated in the tenth exon of *BRF2*, while *fes1a-2 brf2-ed2* lost 7 base pairs in the tenth exon of *BRF2* (**Figure 2A**). To determine their thermotolerance, 10-day-old *Arabidopsis* seedlings were first exposed to 38°C for 2 h, then subjected to lethal heat treatment at 45°C for 2 h, and finally moved to a room at 23°C for 7 days. As shown in **Figure 2B** and **Supplemental Figure 5**, mutations in *BRF2* increased the thermotolerance of *fes1a-2* and the accumulation of HSPs. Interestingly, the *brf2* single mutant did not exhibit any visible change in thermotolerance (**Supplemental Figure 6**). *Arabidopsis BRF* gene family has three members, *BRF1* (*At2g45100*), *BRF2* (*At3g09360*), and *BRF3* (*At2g01280*). We further bred the double mutants of BRFs. The homogenous double mutants of *brf1 brf3* and *brf2 brf3* were generated, while the double mutation of BRF1 and BRF2 led to sterility (Zhang et al., 2019). We determined the thermotolerance of *brf1 brf3* and *brf2 brf3.* Both presented increases of thermotolerance, compared to WT (**Supplemental Figure 7**), suggesting that BRF not only acts as a suppressor of the *fes1a* mutant but also negatively regulates the thermotolerance in WT.

We further explored the effect of BRF2 on thermotolerance by overexpressing HA-tagged BRF2 under the control of an *HSP* promoter in both WT plants and *fes1a-2* mutants. Of the T_3_ transgenic lines, *OEBRF2-1/WT* and *OEBRF2-2/WT*, which only had one HA-tagged BRF2 insertion, were selected for further studies. We crossed the *OEBRF2/WT* lines with *fes1a-2* to obtain the allelic *OEBRF2-1*/*fes1a-2* and *OEBRF2-2*/*fes1a-2* lines. Western bolt found that BRF2 was overexpressed in transgenic lines (**Figure 2C**). BRF2 overexpression lines were subjected to 38°C for 2 h acclimation, then 45°C for 1.5 h lethal heat stress, and finally left at 23°C for 7 days. We found that overexpression of *BRF2* led to increased susceptibility of *fes1a-2* to heat stress, but had no effect on the WT background (**Figure 2D**). Taken together, our results show that the abundance of BRF2 in *fes1a* mutants was correlated with the sensitivity of *fes1a* mutants to heat stress, further suggesting that *BRF2* is a negative regulator of thermotolerance defect in *fes1a*.

*BRF1*, *BRF*2, and *BRF3* may have similar functions. To test this speculation, *fes1a-1 brf1* and *fes1a-1 brf3* double mutants were generated by crossing *fes1a-1* with *brf1* and *brf3* T-DNA insertion mutants (**Figure 2E**), respectively, and the thermotolerance of the double mutants was evaluated. As shown in **Figure 2F**, knockout of *BRF1* or *BRF3* significantly increased the thermotolerance of *fes1a-1*. Therefore, our results suggest that all three *BRF* genes have similar functions in regulating the heat sensitivity of *fes1a* mutants.

**Figure 3:**
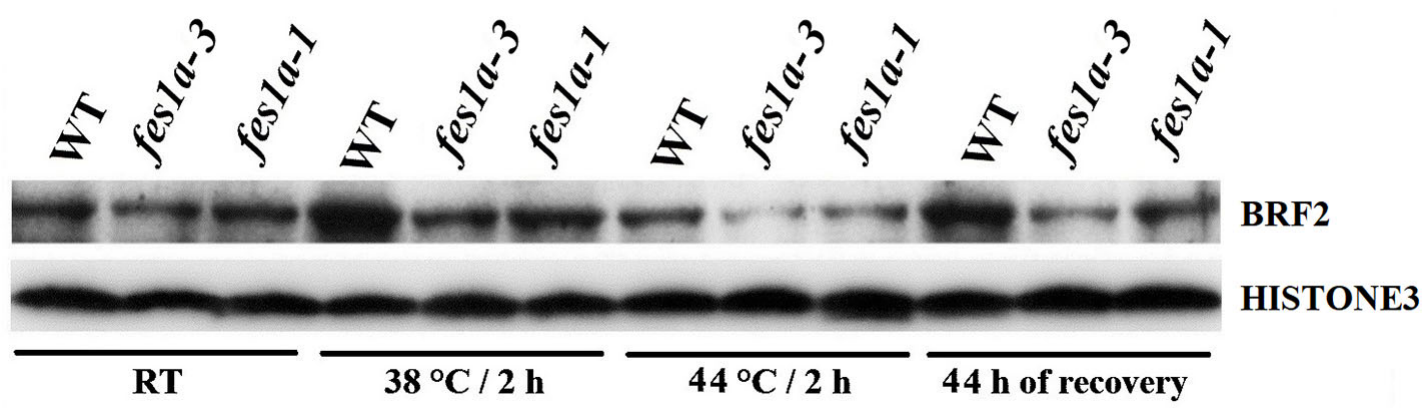
Western blot results exhibiting the expression of BRF2 protein in 10-day-old seedlings of WT and *fes1a* mutants.

We determined the level of BRF2 in *fes1a-1* and *fes1a-3* by using the anti-BRF2 antibody that identified BRF2 protein well in spite of a very faint nonspecific background signal included (**Supplemental Figure 8**). Western blot showed the amount of BRF2 in *fes1a-1* was higher than that in *fes1a-3* (**Figure 3**), which supported the suggestion that BRF2 negatively regulated thermotolerance in *fes1a* mutants.

### *Arabidopsis* BRF2 Is a Functional Ortholog of Yeast BRF1

**Figure 4:**
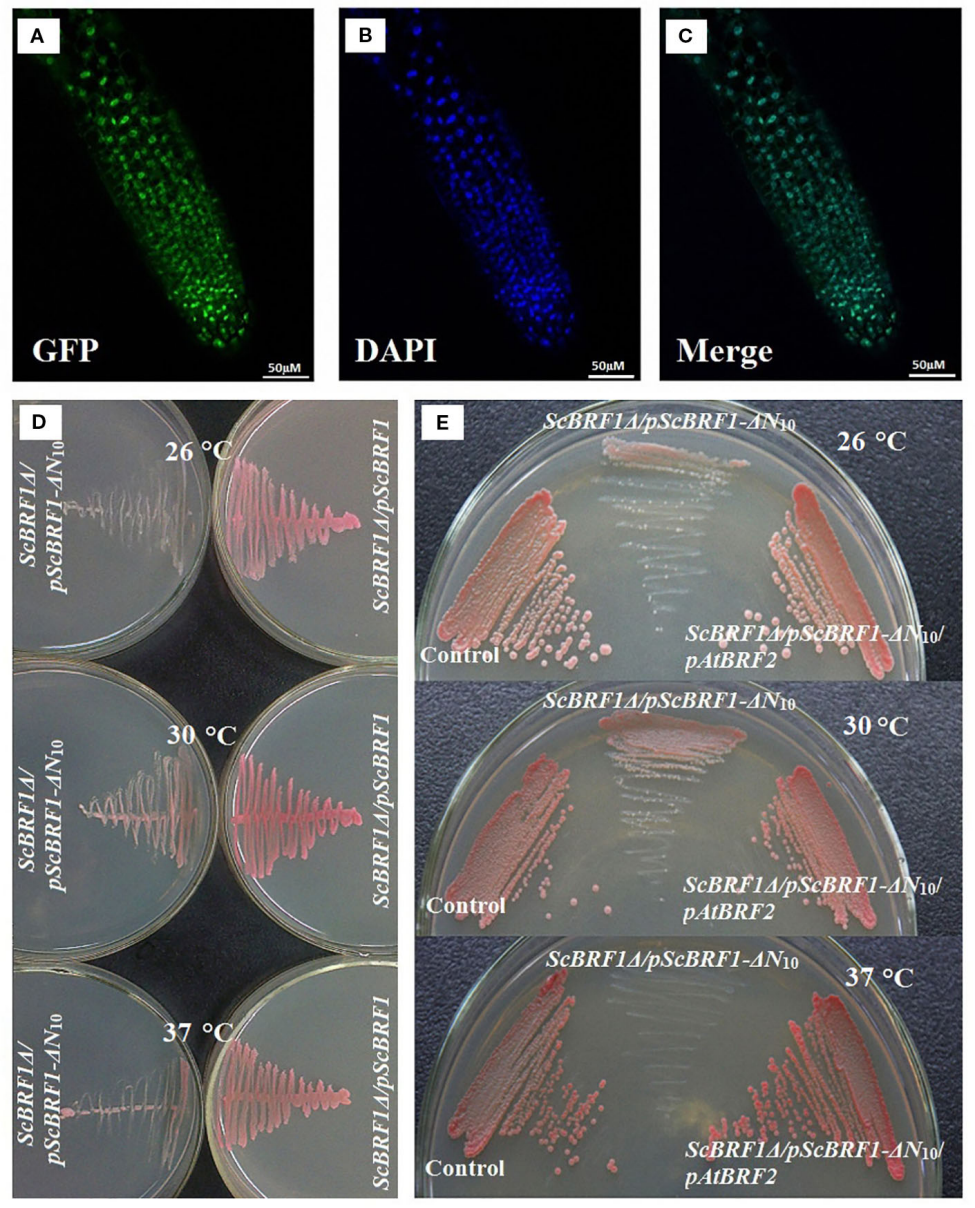
*Arabidopsis BRF2* complements the thermotolerance defect of *S. cerevisiae brf1* mutant. **(A–C)** Confocal images illustrating subcellular localization of the BRF2-GFP fusion protein in *Arabidopsis* roots. The green fluorescent image of BRF2-GFP fusion **(A)** and the blue fluorescent of nucleus stained with DAPI **(B)** are merged into **(C)**. **(D)** The substitution of ScBRF1 with an N-terminal truncated fragment (harbored in pScBRF1-ΔN10 plasmid) resulted in defect in growth, especially at adverse temperatures. **(E)** The susceptibility of ScBRF1Δ/pScBRF1-ΔN10 strain to adverse temperatures was remedied by overexpression of the *Arabidopsis BRF2*. W303 strain with pJFE3 and pYX242WS vectors was used as a control.

Phylogenetic analysis found that BRFs from yeasts, plants, and animals clustered together, suggesting that they all evolved from a common ancestor (**Supplemental Figure 9**, **Supplemental Table 9**). Subcellular localization analysis showed that the fusion protein BRF2-GFP was localized in the nucleus (**Figures 4A–C**). To investigate whether BRF2 is a functional ortholog of *Saccharomyces cerevisiae* BRF1, we performed yeast functional complementation experiments. As knock-out of *ScBRF1* is fatal (Colbert and Hahn, 1992), we adopted an alternative procedure to obtain a *ScBRF1* conditional knockout mutant. The W303a strain was first transformed with the plasmid *ScBRF1-ΔN_10_*-pYX (**Supplemental Table 3**), in which open reading frame of *ScBRF1* was deleted 30 bp from the start codon. Subsequently, the *ScBRF1* locus was knocked out to generate *ScBRF1Δ/pScBRF1-ΔN*_10_ strain. Under normal conditions, *ScBRF1Δ/pScBRF1-ΔN*_10_ strain grew, albeit slowly (**Figure 4D**). Under high and low temperature stresses, the growth of strain *ScBRF1Δ/pScBRF1-ΔN*_10_ was further severely suppressed (**Figure 4D**). This defect in thermotolerance was rescued by expressing *AtBRF2* in *ScBRF1Δ/pScBRF1-ΔN*_10_ strain (**Figure 4E**, **Supplemental Table 3**), suggesting that the *Arabidopsis BRF2* is a functional ortholog of yeast *BRF1*.

### At the Recovery Stage, HSP Accumulation Is Different Between the *fes1a* Mutants

**Figure 5:**
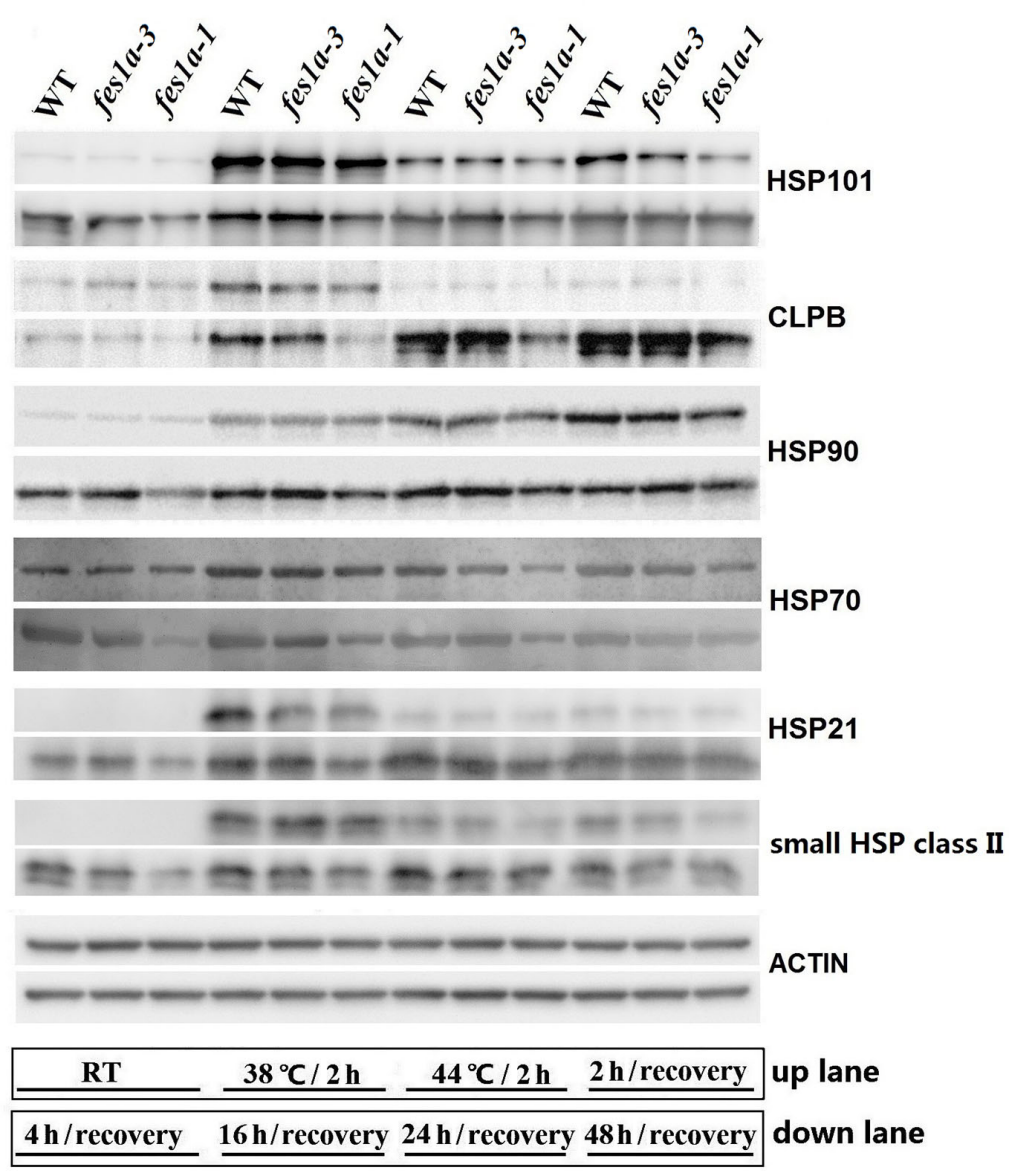
The expression of HSPs over the period from heat acclimation to the stage of recovery. Ten-day-old seedlings were first acclimated for 2 h at 38 °C, and then exposed to 44 °C for 2 h, and lastly returned to room temperature (RT) for recovery. At the indicated times (shown at the bottom of the picture), proteins were extracted for western blots. ACTIN was used as a loading control.

Of the three *fes1a* mutants examined in this study, *fes1a-1* and *fes1a-3* were the most and least sensitive to heat stress, respectively, although *fes1a-1* showed slightly sensitive to heat stress, compared to *fes1a-2*. We chose *fes1a-1* and *fes1a-3* to detect protein expression changes in response to heat stress. Western blot results (**Figure 5**) showed that all examined HSPs, including HSP101, CLPB, HSP90, HSP70, HSP21, and small HSP class II were at similar levels in *fes1a-1* and *fes1a-3* after heat acclimation at 38°C for 2 h, which hardly explain the differential thermotolerance of these two *fes1a* mutants. Therefore, we further determined the levels of HSP proteins over 48 h of the recovery period. In general, the levels of the HSPs at the recovery stage were in the order of WT *> fes1a-3 > fes1a-1*, and the differences were significant, especially as for HSP101, CLPB, HSP70, and small HSP (**Figure 5**). These results suggest that accumulation of HSPs at the recovery stage was well correlated with the differential thermotolerance among WT, *fes1a-1*, and *fes1a-3*.

### The Mutant of *fes1a-3* Presented Higher Activity of RNA Pol II than *fes1a-1*

**Figure 6:**
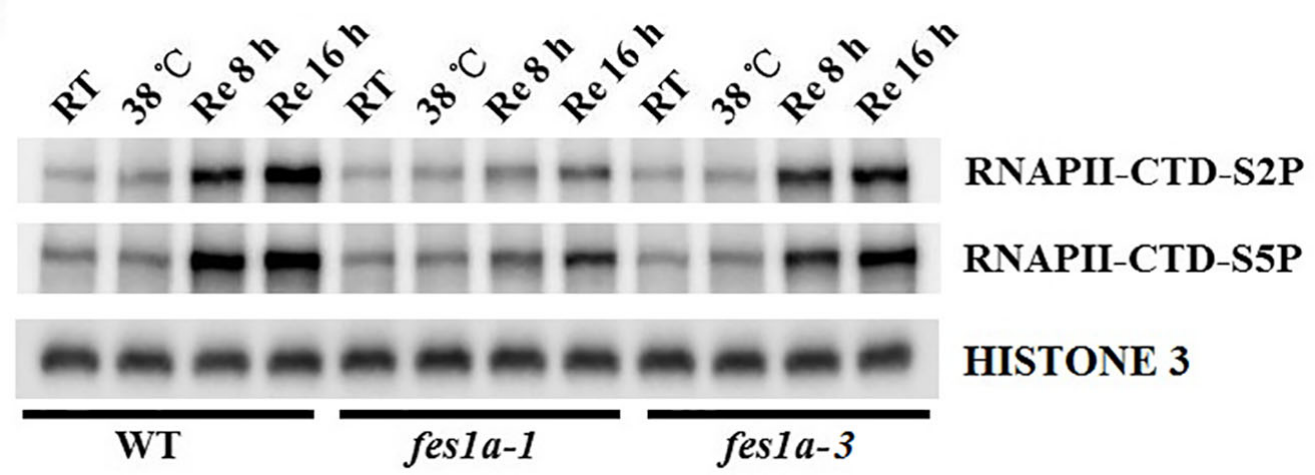
Phosphorylation levels of the C-terminal domain (CTD) of RNA Polymerase (Pol) II largest subunit 1 (RPB1) in *fes1a-1* and *fes1a-3.* Ten-day-old seedlings were acclimated for 2 h at 38 °C, then exposed to 44 °C for 2 h, and lastly returned to room temperature for recovery. Seedlings were sampled at indicated times. Phosphorylation levels of RPB1 CTD repeats were determined with RNA Pol II CTD repeat phospho Ser-2 and Ser5 antibodies.

According to the different expressions of HSPs at the recovery stage, we further evaluated the transcriptional activity of RNA Pol II in *fes1a-1* and *fes1a-3* by examining the phosphorylation of the C-terminal domain (CTD) of RNA Polymerase II largest subunit 1 (RPB1), using anti-S2P-CTD and anti-S5P-CTD antibodies. The CTD of RPB1 is composed of tandem heptad repeats that are critical for the activity of RNA Pol II (Hsin and Manley, 2012). The phosphorylation status of Ser2 and Ser5 in the CTD of RPB1 could serve as an indicator of RNA Pol II activity. Although lower than in WT, the level of phosphorylated RPB1 CTD in *fes1a-3* was higher than in *fes1a-1* at the recovery stage after exposure to a severe heat stress (**Figure 6**). These results confirm that the activity of RNA Pol II increased faster in *fes1a-3* than *fes1a-*1 during recovery from a severe heat shock. Further, we determined the activity of RNA Pol II in WT, *brf2*, *fes1a-2* and *fes1a-2 brf2-ed1* double mutant. The result indicated that the single mutant of *brf2* did not change the activity of RNA Pol II, compared to WT. A mutation of *BRF2* in genetic background of *fes1a-2,* however, increased phosphorylation levels of RPB1 CTD at the recovery stage after a severe heat stress (**Supplemental Figure 10**), suggesting that BRF2 negatively affects the activity of RNA Polymerase II in *fes1a*.

## Discussion

In this study, we found that three T-DNA insertion mutants of *fes1a* exhibited significantly different heat sensitivities (**Figure 1**). The closer the T-DNA insertion was towards the 3′UTR of *FES1A*, the more tolerant the *fes1a* mutant was to heat stress. Using allelic hybridization (**Supplemental Table 8**) and evaluation of double mutants, we found that *BRF2*, a gene downstream of *FES1A,* is responsible for increasing the thermo-sensitivity of *fes1a* mutants. Knockout of *BRF2* in a *fes1a* mutant rescued the *fes1a* thermotolerance defect, while overexpression of *BRF2* in *fes1a* mutant further increased heat sensitivity (**Figure 2**). Thus, the lower level of BRF2 in *fes1a-3* was suggested to being responsible for its more significant decrease of sensitivity to heat stress (**Figure 3**) when compared to *fes1a-1.*

**Figure 7:**
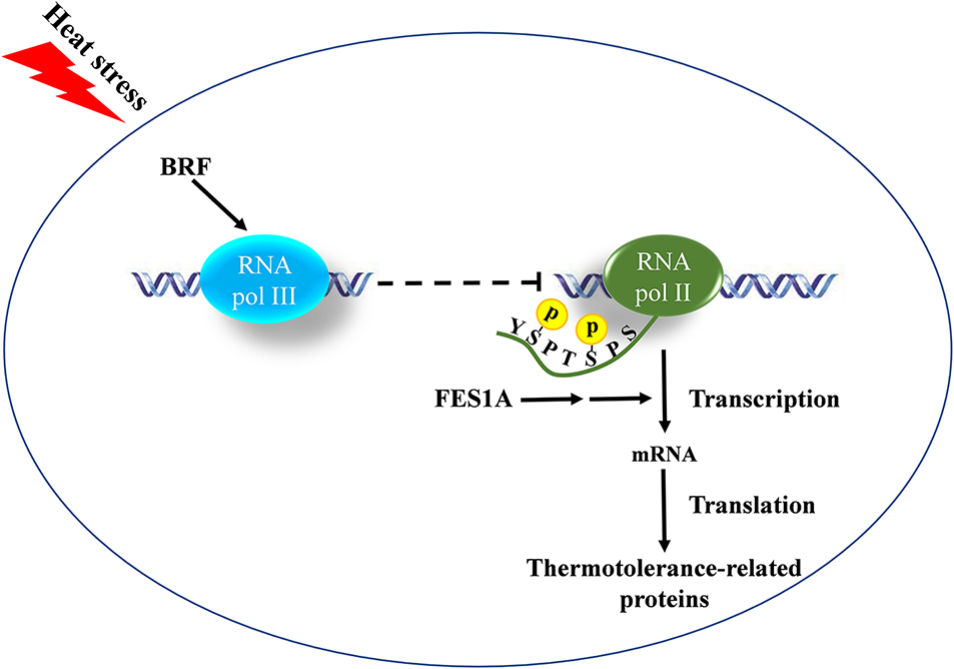
The possible relationships of FES1A, BRF, and thermotolerance-related genes. BRF negatively regulates the transcriptional strength of RNA Pol II by the cross-talking between RNA Pol III and II, which is indicated by the phosphorylation level change of the C-terminal domain (CTD) of RNA Polymerase (Pol) II largest subunit 1 (RPB1). The negative regulation is magnified by the mutation of FES1A and affects thermotolerance-related protein levels.

*Arabidopsis* BRF2 was a negative regulator of RNA pol II transcription in the *fes1a* mutant. The amount of BRF2 in *fes1a* mutants was negatively correlated with the activity of RNA Pol II. RPB1 is the largest subunit of the RNA Pol II complex (Hsin and Manley, 2012). At transcription initiation, Ser2 and Ser5 residues in the CTD of RPB1 are phosphorylated, whereas as transcription nears termination, Ser5-P and Ser2-P are gradually dephosphorylated, regenerating unphosphorylated RPB1 that can be recycled for another round of transcription. Thus, the level of phosphorylated CTD reflects the activity of Pol II transcription. As suggested by a significant increase in the phosphorylated status of Ser2 and Ser5 of the CTD of RPB1 in *fes1a-3* (**Figure 6**), RNA Pol II transcription is more active in *fes1a-3* than in *fes1a-1*. In addition, knockout of the *BRF2* increased the extent of phosphorylation of RPB1 in *fes1a-2* (**Supplemental Figure 10**), suggesting BRF2 negatively regulated the strength of Pol II transcription in *fes1a*. Further, western blot results showed that the accumulation of HSPs in *fes1a-3* was higher than in *fes1a-1.* Therefore, the differential strengths of general transcription and the differential accumulation of HSPs are molecular evidence of significant differences in thermosensitivities between *fes1a-1* and *fes1a-3*. The possible relationships among FES1A, BRF, and thermotolerance-related genes are presented in **Figure 7**.

It has been shown that the mutation of *S. cerevisiae BRF1* affected RNA Pol II transcription. Mutations in *ScBRF1-ΔN10*, *ScBRF1-ΔN25*, and *ScBRF1-ΔC50* all lead to an increased expression of cytochrome c (Colbert and Hahn, 1992), while the yeast mutant *ScBRF1-II.6*, which has point mutations at D464A and D466A on *ScBRF1*, enhances mRNA transcription (Conesa et al., 2005). In short, the mutation of ScBRF1 is in favor of Pol II transcription. Our results suggest that *Arabidopsis* BRF2, a functional ortholog of yeast ScBRF1, negatively affects the activity of RNA Pol II and is involved in the heat response of *fes1a* mutants, indicating there is a cross-talking between RNA Pol III and Pol II, which affects thermotolerance. Further, the negative regulation of the double mutations of BRFs on thermotolerance in WT suggests that the cross-talking is independent of *fes1a*. The single mutation of BRF affected the thermotolerance in *fes1a*, but the thermotolerance in WT was regulated by the double mutations of BRFs. In other words, the effect of BRF is magnified in *fes1a.* However, the involved mechanism needs to be studied further.

## Data Availability Statement

The raw data supporting the conclusions of this manuscript will be made available by the authors, without undue reservation, to any qualified researcher.

## Author Contributions

CF, XLiu and XLi performed majority of the research. Western blot was performed by CF, XLiu, PH, and JG. WY and JZ performed the phenotyping experiments. Bioinformatic analysis was carried out by XLiu and LZ. JL designed the research and wrote the main manuscript text. JL, CF, CM, and DZ revised the manuscript. All authors read and approved the final manuscript. CF, XLiu, and XLi contributed equally to this work.

## Funding

This work was supported by the National Natural Science Foundation of China (31270298, 31470352, 3182004) and the Research Fundamental Capacity Improvement Project for Middle Age and Youth Teachers of Guangxi Universities (2019KY0517). Research in the lab of DZ was supported by the National Science Foundation grant IOS-1322796 and the Research Growth Initiative (RGI). The funder had no role in the study design, data collection and analysis, decision to publish, or preparation of the manuscript.

## Conflict of Interest

The authors declare that the research was conducted in the absence of any commercial or financial relationships that could be construed as a potential conflict of interest.
