# Supplementary material for: BRF Negatively Regulates Thermotolerance Defect of fes1a in Arabidopsis
Source: Front Plant Sci. 2020 Mar 10;11:171. doi: 10.3389/fpls.2020.00171 (PMC7077510; doi:10.3389/fpls.2020.00171)
Supplement: Supplementary file 1 [file DataSheet_1.docx]

Supplementary Material

**Supplementary Figures**

**Figure legends**

**Supplementary Figure 1.** PCR identification of *fes1a-1* to *fes1a-3* mutants

**Supplementary Figure 2.** Western blot analysis of FES1A in wild-type and *fes1a-1* to *fes1a-3* mutants.

**Supplementary Figure 3.** The survival rates of the seedlings correlated to Figure 1D.

**Supplementary Figure 4.** Relative expression levels of BRF2 transcript in WT and *fes1a* mutants.

**Supplementary Figure 5.** Differential expression of HSPs proteins in wild-type, *fes1a-2* mutant and the *fes1a-2 brf2-ed1* double mutant.

**Supplementary Figure 6.** Evaluation of thermotolerance of *brf* mutants.

**Supplementary Figure 7.** Evaluation of thermotolerance of double *brf* mutants.

**Supplementary Figure 8.** The determination of immunological recognition of anti-BRF2 antibody.

**Supplementary Figure 9.** Phylogenetic tree of BRF2-like proteins.

**Supplementary Figure 10.** Phosphorylation levels of the C-terminal domain (CTD) of RNA Polymerase (Pol) II largest subunit 1 (RPB1) in wild-type, *brf2*, *fes1a-2* and *fes1a-2 brf2-ed1* double mutant*.*

**
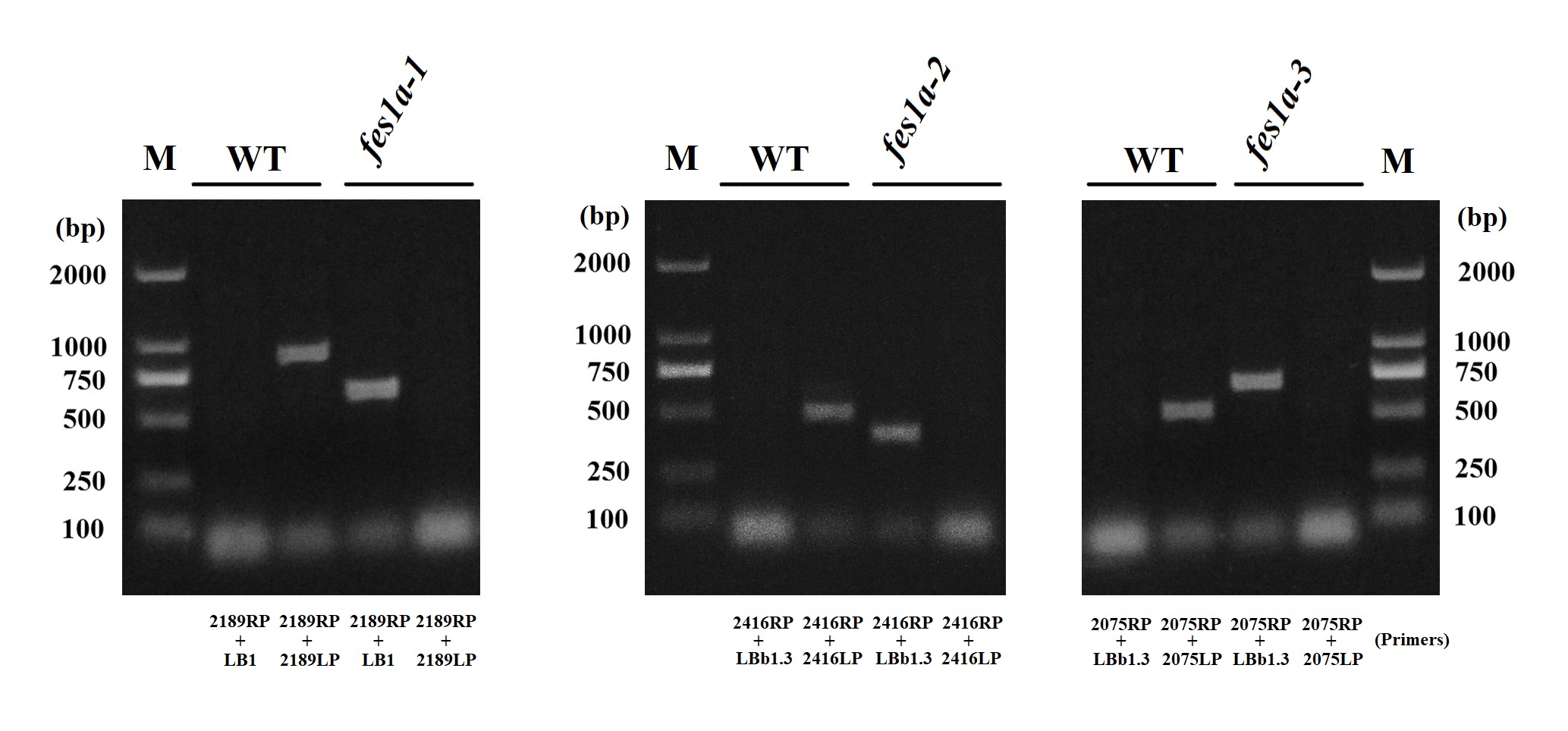
**

**Supplementary Figure 1.** PCR identification of *fes1a-1* to *fes1a-3* mutants. The primers used are listed in Supplementary Table 2.





**Supplementary Figure 2.** Western blot analysis of FES1A in wild-type and *fes1a-1* to *fes1a-3* mutants. Plants were exposed to 38 °C for 2 h before sampling for western blots. RUBISO-L was used as a loading control.


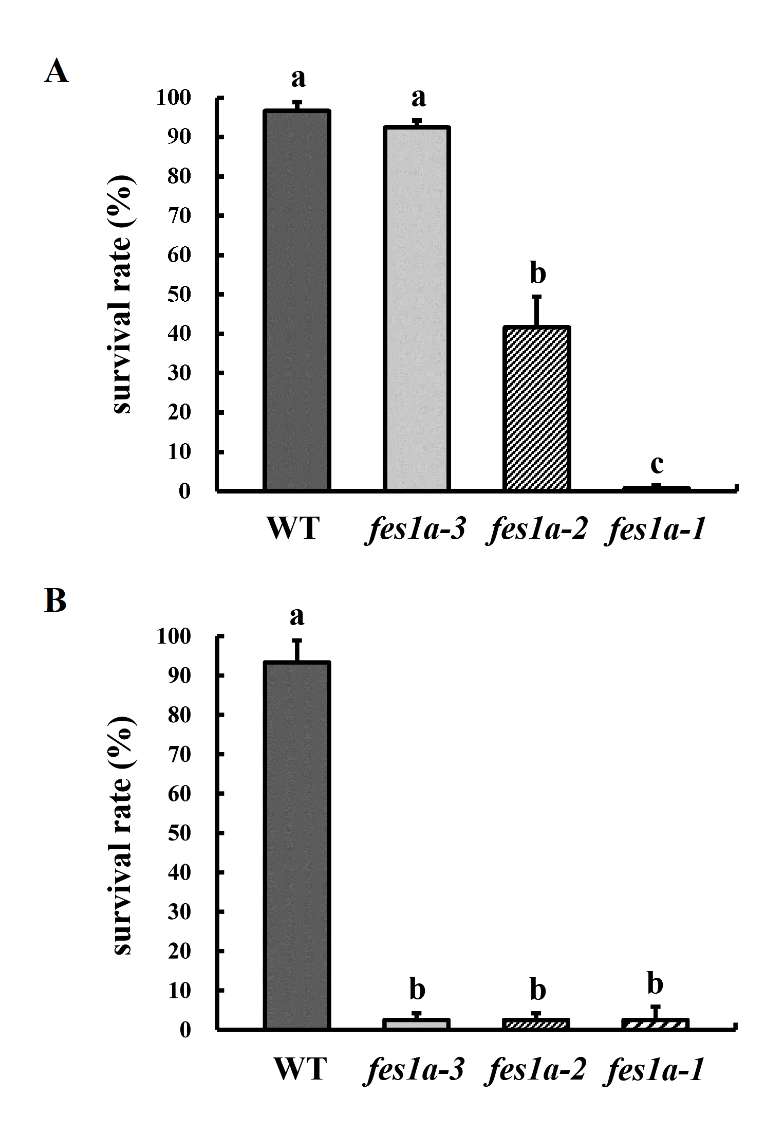


**Supplementary Figure 3.** The survival rates of the seedlings correlated to Figure 1D. **(A)** The survival rate correlated to the left petri dish of Figure 1D. **(B)** The survival rate correlated to the right petri dish of Figure 1D. All Data represent the means of 3 replicates ± SD. For each column, different letters *a*, *b*, *c* indicate significant differences at *P* < 0.05.


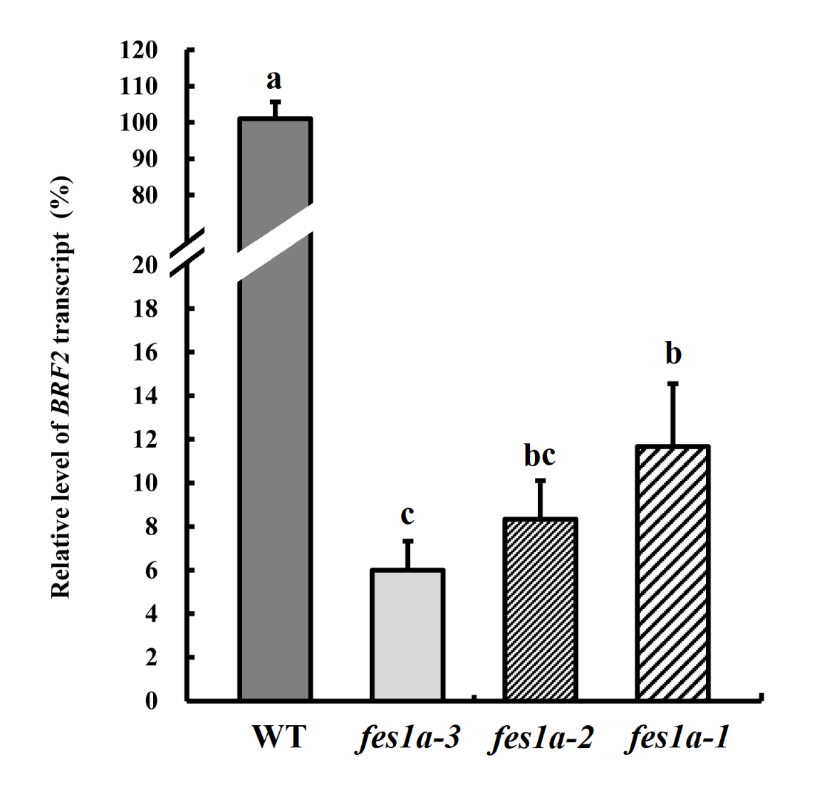


**Supplementary Figure 4.** Relative expression levels of BRF2 transcript in WT and *fes1a* mutants. After the plants were treated at 38 °C for 2 h, total RNA was isolated and then used for quantitative real-time PCR analysis. All Data represent the means of 3 replicates ± SD. For each column, different letters a, b, c indicate significant differences at *P* < 0.05.


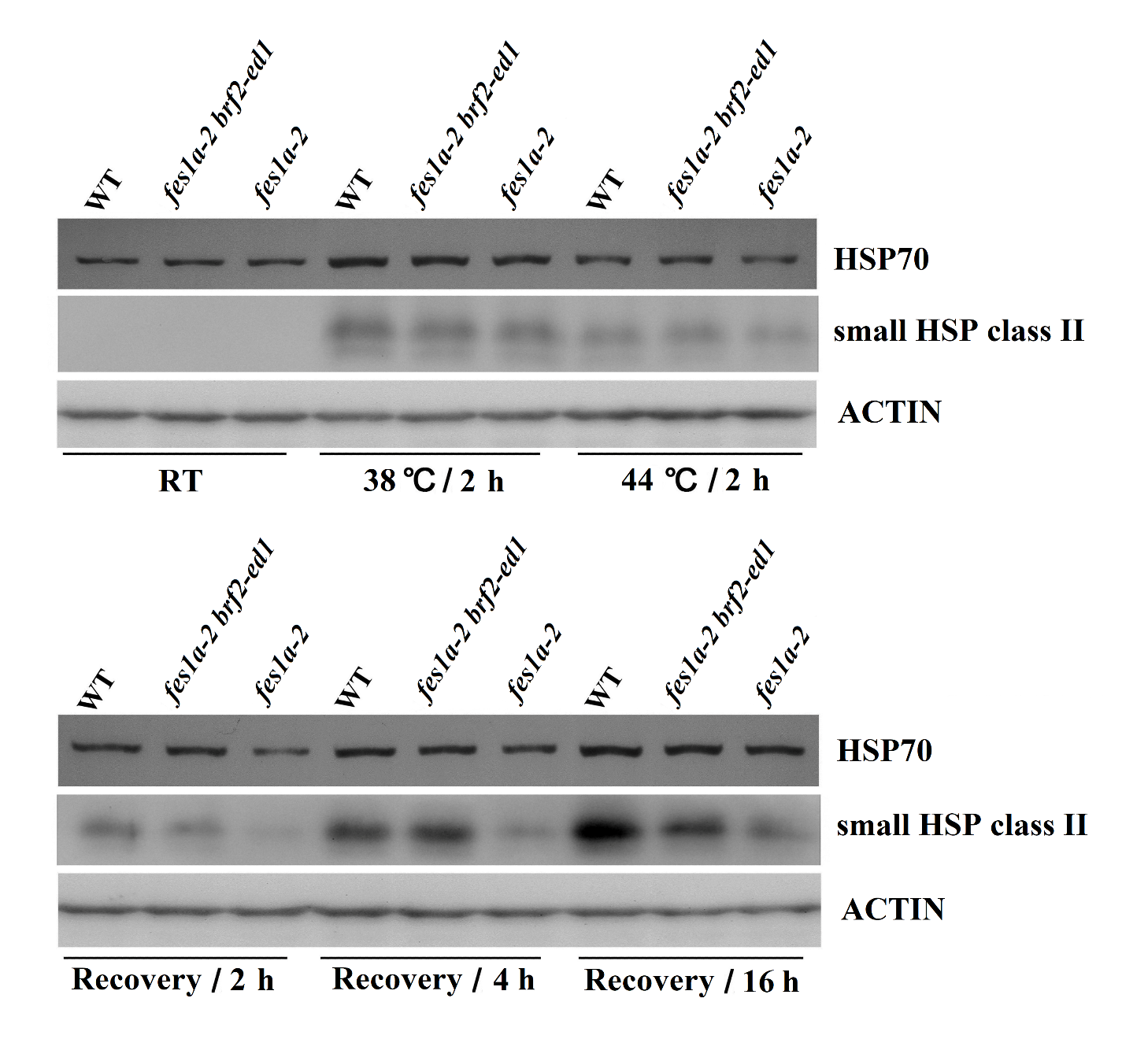


**Supplementary Figure 5.** Differential expression of HSPs proteins in wild-type, *fes1a-2* mutant and the *fes1a-2 brf2-ed1* double mutant. The temperature regime of heat stress was the same with that in Figure 2B.


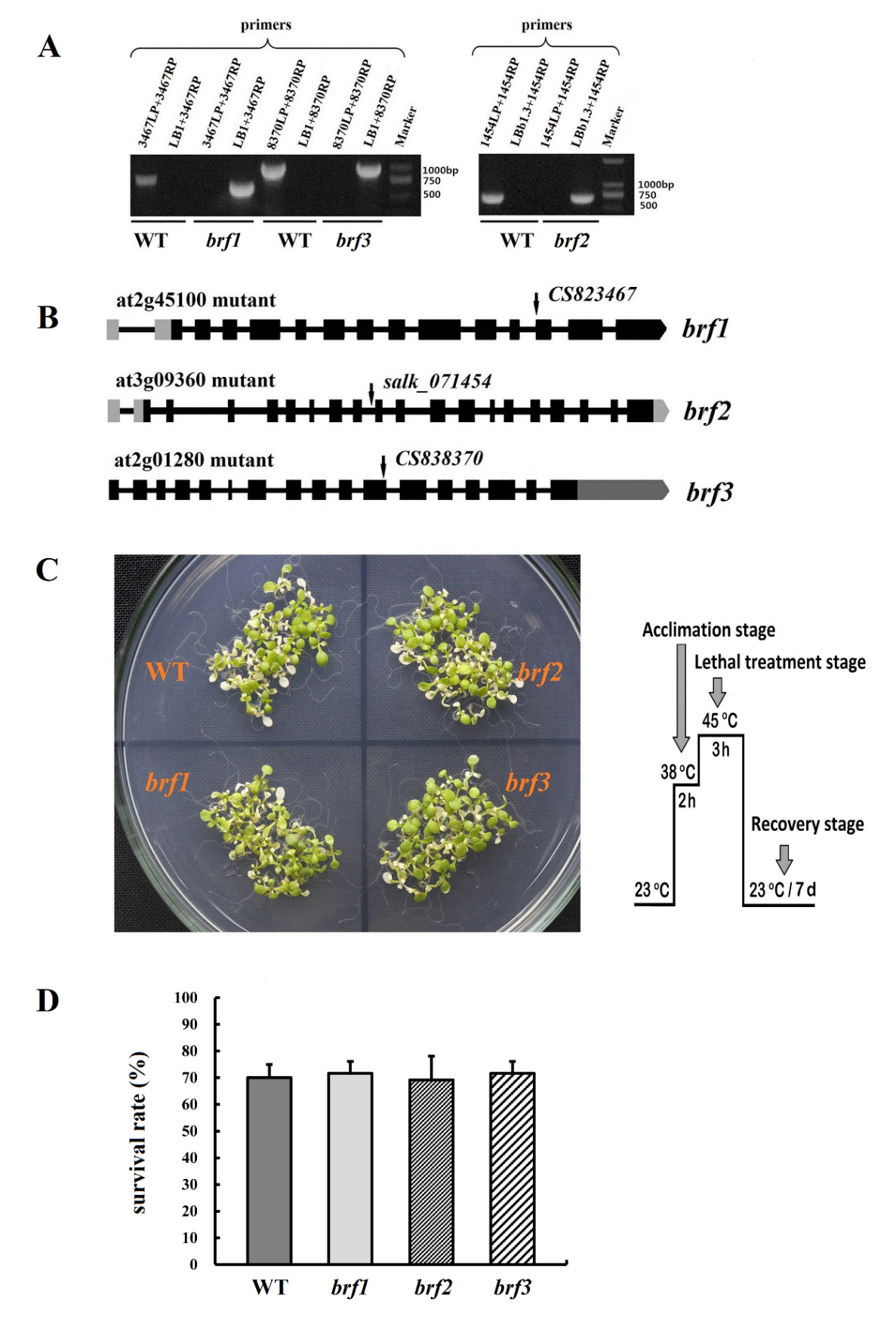


**Supplementary Figure 6.** Evaluation of thermotolerance of *brf* mutants. **(A)** Identification of *brf* mutants by PCR. **(B)** T-DNA insertion sites in *brf* mutants. **(C)** The mutation of *BRF1*, *BRF2*, or *BRF3* had no effect on acquired thermotolerance in *Arabidopsis*. The temperature regime used for thermotolerance evaluation is shown on the left. The phenotype picture was taken after 7 days of recovery. **(D)** The survival rates of seedlings correlated to **(C)**. Data represent the means of 3 replicates ± SD.


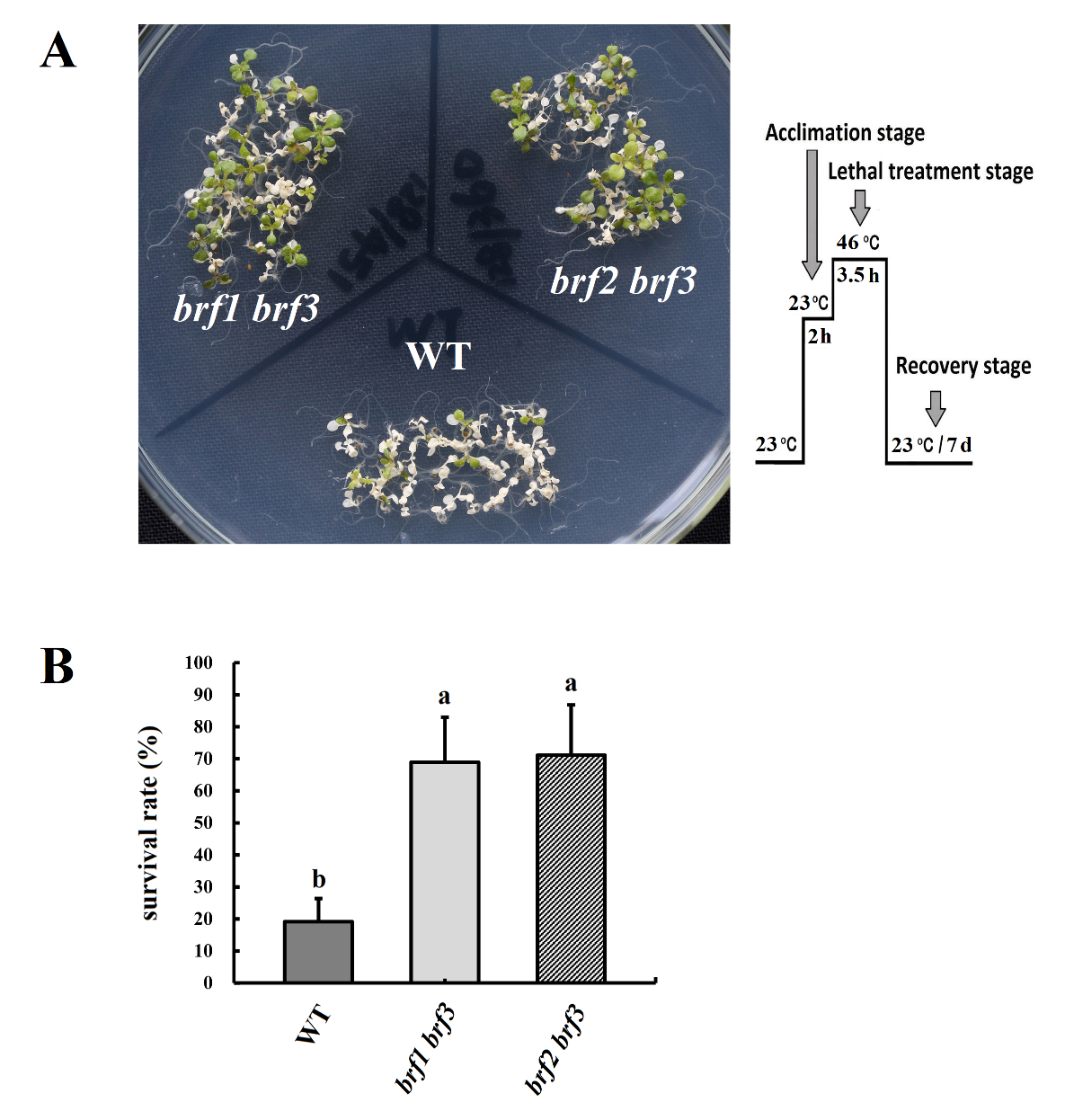


**Supplementary Figure 7.** Evaluation of thermotolerance of double *brf* mutants. **(A)** Seedlings were subjected to a heat acclimation and then a lethal heat stress. The phenotype picture was taken after 7 days of recovery. The temperature regime used for thermotolerance evaluation is shown on the right. **(B)** The survival rates of seedlings correlated to **(A)**. Data represent the means of 3 replicates ± SD. For each column, different letters *a*, *b*, *c* indicate significant differences at *P* < 0.05.


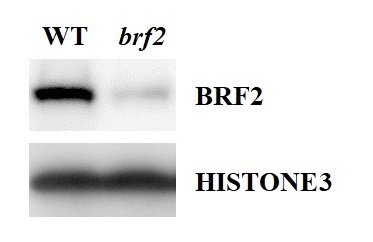


**Supplementary Figure 8.** The determination of immunological recognition of anti-BRF2 antibody. The total proteins from WT and *brf2* were used for western-blotting experiment, in which the anti-BRF2 antibody was used as the primary antibody.


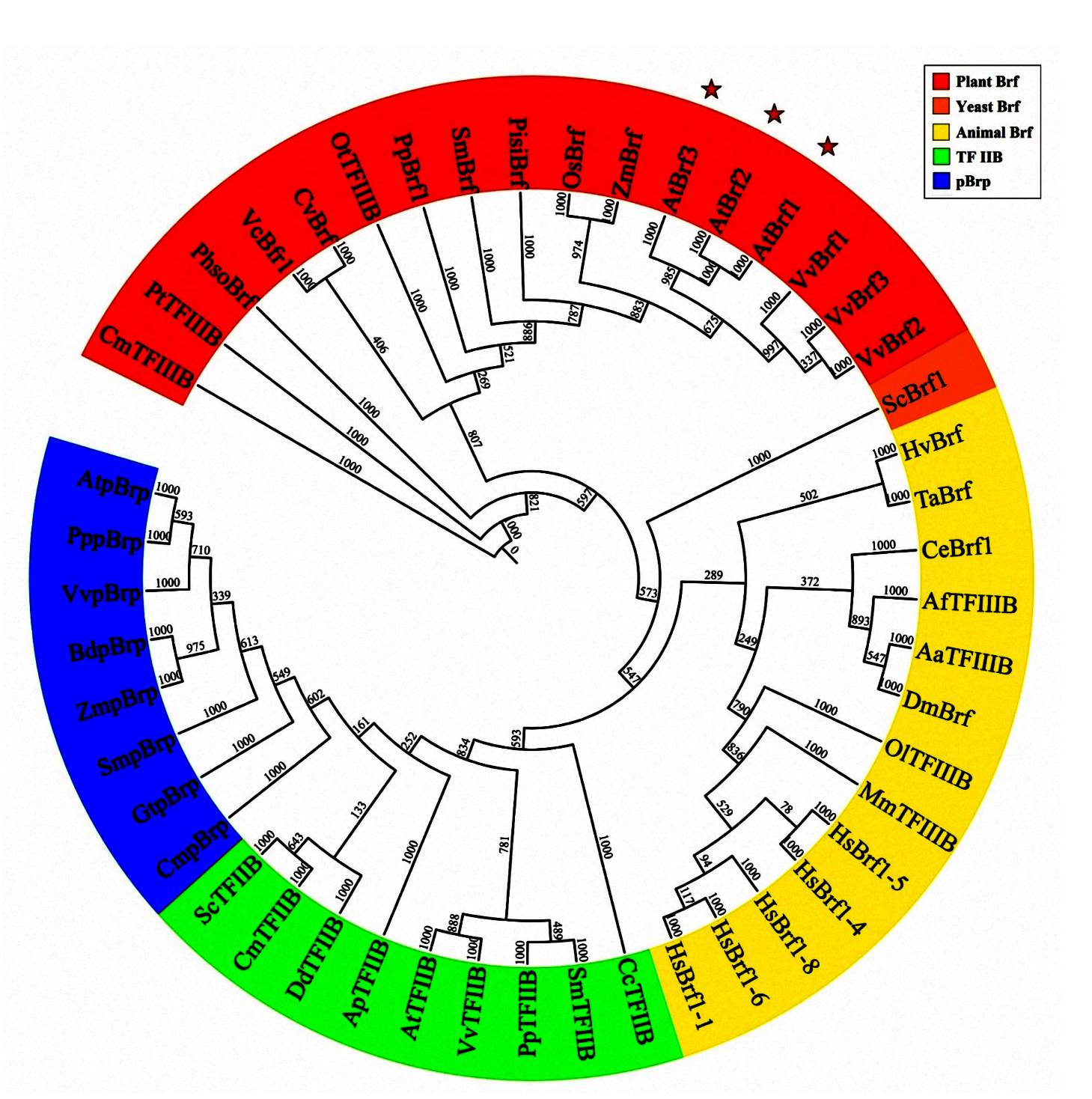


**Supplementary Figure 9.** Phylogenetic tree of BRF2-like proteins. The unrooted tree was constructed by using the maximum likelihood method with the software, PHYLIP3.68, and the reliability was estimated from 1,000 bootstrap replicates. The numbers on the branches indicate the consensus values. Interactive Tree of Life (iTOL) was used to visualize the constructed phylogenetic tree. GenBank accession numbers of BRF2-like proteins are presented in Supplementary Table 9.


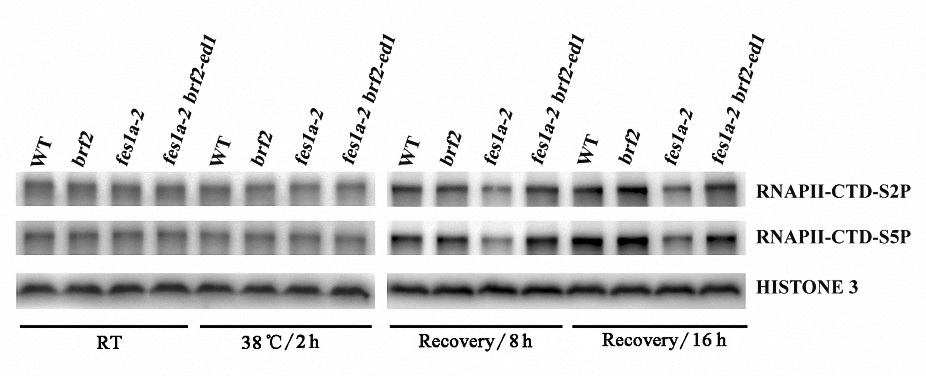


**Supplementary Figure 10.** Phosphorylation levels of the C-terminal domain (CTD) of RNA Polymerase (Pol) II largest subunit 1 (RPB1) in wild-type, *brf2*, *fes1a-2* and *fes1a-2 brf2-ed1.* Ten-day-old seedlings were acclimated for 2 h at 38 °C, then exposed to 44 °C for 2 h, and lastly returned to room temperature for recovery. Seedlings were sampled at indicated times. Phosphorylation levels of RPB1 CTD repeats were determined with RNA Pol II CTD repeat phospho Ser-2 and Ser5 antibodies.

**Supplementary Tables**

**Table legends**

**Supplementary Table 1.** Arabidopsis mutant lines used in this study.

**Supplementary Table 2.** Primer sequences used for identification of mutants.

**Supplementary Table 3.** *Saccharomyces cerevisiae* strains and plasmids used in the present study.

**Supplementary Table 4.** Primer sequences used for yeast functional complementation analysis.

**Supplementary Table 5.** Primer sequences used for vector construction of transgenic *Arabidopsis* lines.

**Supplementary Table 6.** Primer sequences used for RT-PCR.

**Supplementary Table 7.** Primer sequences used for qRT-PCR.

**Supplementary Table 8.** The correlation of heat susceptibilities between parents and homozygote offspring that were bred from allelic mutant hybridization.

**Supplementary Table 9.** The species used for constructing the phylogenetic tree and accession numbers in Genbank.

**Supplementary Table 1.** *Arabidopsis* mutant lines used in this study.

| Gene_id | Gene names | Stock names | Brief name in this paper |
| --- | --- | --- | --- |
| *At3g09350* | *FES1A* | *cs842189* | *fes1a-1* |
|  |  | *salk_012416* | *fes1a-2* |
|  |  | *salk_072075* | *fes1a-3* |
| *At2g45100* | *BRF1* | *cs823467* | *brf1* |
| *At3g09360* | *BRF2* | *salk_071454* | *brf2* |
| *At2g01280* | *BRF3* | *cs838370* | *brf3* |

**Supplementary Table 2.** Primer sequences used for identification of mutants.

| Gene name | Mutant names | Stock names | Primer | Primer sequence (5’ to 3’) |
| --- | --- | --- | --- | --- |
| *FES1A*  (*At3g09350*) | *fes1a-1* | *cs842189* | 2189LP | caagttcctgacttctaggattgttc |
|  |  |  | 2189RP | gtacggacattcatcttgtagttgg |
|  | *fes1a-2* | *salk_012416* | 2416LP | caagtaatccacgaagagcggc |
|  |  |  | 2416RP | attcacgctcgaacccaggc |
|  | *fes1a-3* | *salk_072075* | 2075LP | gtagcaaatgctccacagtaaatcc |
|  |  |  | 2075RP | tgccaatggttatgctggcctaag |
| *BRF1* (*At2g45100*) | - | *cs823467* | 3467LP | gaccatgctatcaacagcttctatg |
|  |  |  | 3467RP | ggattatgtgagagctgttacgatg |
| *BRF2* (*At3g09360*) | - | *salk_071454* | 1454LP | ccagaatccgtatcgccaaactc |
|  |  |  | 1454RP | gaaatgctgtaccttgtggaaaatcg |
| *BRF3* (*At2g01280*) | - | *cs838370* | 8370LP | ccgaggctggaagtttaactg |
|  |  |  | 8370RP | gcaattagcagccttcagagc |
| SALK_T-DNA |  |  | LBb1.3 | gattttgccgatttcggaaccac |
| SAIL_T-DNA |  |  | LB1 | cagaaatggataaatagccttgcttcc |

**Supplementary Table 3.** *Saccharomyces cerevisiae* strains and plasmids used in the present study.

| Strain | Relevant features/genotype | Source/reference |
| --- | --- | --- |
| W303a | *MATa leu2-3/112 ura3-1 trp161 his3-11/15 ade2-1 can1-100 GAL SUC2 mal0* | Lab store |
| Control | W303a, pJFE3*/*pYX242WS | This work |
| W303a/ *pScBRF1-ΔN10* | W303a, *pScBRF1-ΔN10*-pYX | This work |
| *ScBRF1Δ*/*pScBRF1-ΔN10* | W303a, *ScBRF1::KanMX4/ pScBRF1-ΔN10*-pYX | This work |
| *BRF1Δ*/*pScBRF1-ΔN10/pAtBrf2* | W303a, *ScBRF1::KanMX4/pScBRF1-ΔN10*-pYX*/* *pAtBRF2*-pJFE3 | This work |
| Plasmid | Relevant features/genotype | Source/reference^a^ |
| pUG6 | *E. coli* plasmid with segment *LoxP-KanMX4-LoxP* | Güldener et al., 1996 |
| pJFE3 | Yeast 2μm plasmid with *URA3* marker | Shen et al., 2012 |
| pYX242WS | Yeast 2μm plasmid with *LEU2* marker | Wang et al., 2013 |
| *ScBRF1-ΔN10*-pYX | 2μm plasmid, *LEU2*, P*_TEF1_*-*ScBRF1-ΔN10* | This work |
| *AtBRF2*-pJFE3 | 2μm plasmid, *URA3*, P*_TEF1_*-*AtBRF2* | This work |

^a^References:

Güldener U, Heck S, Fielder T, Beinhauer J, Hegemann JH (1996) A new efficient gene disruption cassette for repeated use in budding yeast. Nucleic Acids Res. 24:2519-2524.

Shen Y, Chen X, Peng B, Chen L, Hou J, Bao X (2012) An efficient xylose-fermenting recombinant Saccharomyces cerevisiae strain obtained through adaptive evolution and its global transcription profile. Appl Microbiol Biot 96:1079-1091.

Wang C, Shen Y, Zhang Y, Suo F, Hou J, Bao X (2013) Improvement of L-arabinose fermentation by modifying the metabolic pathway and transport in Saccharomyces cerevisiae. BioMed Res Int 2013:461204.

**Supplementary Table 4.** Primer sequences used for yeast functional complementation analysis.

| Primers | | Sequences (5’ to 3’)^a^ | | Purposes |
| --- | --- | --- | --- | --- |
| pJFE3-ScBRF1-F | cacggatccatggtatggtgtaaccattgcgtga | | To clone *ScBRF1* gene into pJFE3 | |
| pJFE3-ScBRF1-flag-R | gtgctgcagtcacttgtcatcgtcgtccttgtagtcatacaatccaaaatcattaccat | | To clone *ScBRF1* gene into pJFE3 | |
| ScBRF1-upstream-F | gccgtcgctatcaaaaatc | | To delete yeast *ScBRF1* gene | |
| ScBRF1-upstream-R | cttacacactggcattgg | | To delete yeast *ScBRF1* gene | |
| ScBRF1-downstream-F | cgtttaggtaatcaaccctagg | | To delete yeast *ScBRF1* gene | |
| ScBRF1-downstream-R | atctgcgatgagatgag | | To delete yeast *ScBRF1* gene | |
| G418-part1-F | aaaaagagtccatagtcatataccaatgccagtgtgtaagtgcaggtcgacaaccctta | | To delete yeast *ScBRF1* gene | |
| G418-part1-R | tcattcgtgattgcgcctgagc | | To delete yeast *ScBRF1* gene | |
| G418-part2-F | tgcatggttactcaccac | | To delete yeast *ScBRF1* gene | |
| G418-part2-R | tccgttccctttttccttcctagggttgattacctaaacggtggatctgatatcacct | | To delete yeast *ScBRF1* gene | |
| pYX-N10-F | aagaaagcatagcaatctaatctaagttttaattacaaatgccagagagagatctttcc | | To clone *ScBRF1-ΔN10* into pYX242WS | |
| pYX-N10-R | atatcatgcgtagtcaggcacatcatacggatacccgggtcgacttacctaaacaaacc | | To clone *ScBRF1-ΔN10* into pYX242WS | |

^a^Homologous arms and tag sequences were underlined.

**Supplementary Table 5.** Primer sequences for vector construction of transgenic *Arabidopsis* lines.

| Primers | Sequences (5’ to 3’)^a^ | Purposes |
| --- | --- | --- |
| BRF2-pMY72-F | ctccttaagattggaagctgttgt | To clone *BRF2* into pMY72 |
| BRF2-pMY72-R | tcacttaagcaatccaaaatcattacc |  |
| BRF2-HA×2-F | taaggaattcgctttttgagtgaagg | To clone *BRF2-HA*×2 into *P_LeHsp23.8_*-pRT101 |
| BRF2-HA×2-R | caatggtaccgatgatgatggtcgag |  |
| BRF2-DT1-BsF | atatatggtctcgattgtattccactaggcttccggcgtt | To construct CRISPR cassette |
| BRF2-DT1-F0 | tgtattccactaggcttccggcgttttagagctagaaatagc |  |
| BRF2-TD2-BsR | aacgctttccagcgcgcagagaacaatctcttagtcgactctac |  |
| BRF2-TD2-R0 | attattggtctcgaaacgctttccagcgcgcagagaac |  |
| BRF2-GFP-F | gatctcgagctcgaatctgagtttcg | To construct *BRF2-GFP* fusion |
| BRF2-GFP-R | atagaattcaaaatcattaccatcctcttct |  |

^a^Homologous arms were underlined.

**Supplementary Table 6.** Primer sequences for RT-PCR.

| Primers | Sequences (5’ to 3’) | Purposes |
| --- | --- | --- |
| FES1A-RT-F | atggcgaaagacggacctaattgg | For RT-PCR identification of *fes1a* |
| FES1A-RT-R | gtctatggtccaagtagtttcatagg | For RT-PCR identification of *fes1a* |

**Supplementary Table 7.** Primer sequences for qRT-PCR.

| Primers | Sequences (5’ to 3’) | Purposes |
| --- | --- | --- |
| BRF2-qRT-F | gttcgaatcaaggcagtctcac | For qRT-PCR of *BRF2* |
| BRF2-qRT-R | aaacaacagcttccaatcttacggag | For qRT-PCR of *BRF2* |
| ACTIN8-qRT-F | agtggtcgtacaaccggtattgt | For qRT-PCR of *ACTIN8* |
| ACTIN8-qRT-R | gaggatagcatgtggaagtgagaa | For qRT-PCR of *ACTIN8* |

**Supplementary Table 8.** The correlation of heat susceptibilities between parents and homozygous offspring that were bred from allelic mutant hybridization.

| Table S7-1 The phenotypes of F2 population driven from crossing between *fes1a-1* and *fes1a-3*. | | | | |
| --- | --- | --- | --- | --- |
| Genotype of the T-DNA in homozygous offspring | Tested numbers of homozygous offspring | Sorting of phenotypes in offspring, according to their heat susceptible phenotype identical to their parents | | The coefficients of phenotype correlation between parents and homozygote offspring |
|  |  | *fes1a-1* | *fes1a-3* |  |
| *fes1a-1* | 80 | 80 | 0 | 1.00 |
| *fes1a-3* | 78 | 0 | 78 | 1.00 |
| Table S7-2 The phenotypes of F2 population driven from crossing between *fes1a-2* and *fes1a-3*. | | | | |
| Genotype of the T-DNA in homozygous offspring | Tested numbers of homozygous offspring | Sorting of phenotypes in offspring, according to their heat susceptible phenotype identical to their parents | | The coefficients of phenotype correlation between parents and homozygote offspring |
|  |  | *fes1a-2* | *fes1a-3* |  |
| *fes1a-2* | 67 | 67 | 0 | 1.00 |
| *fes1a-3* | 70 | 0 | 70 | 1.00 |

**Supplementary Table 9.** Species used for constructing the phylogenetic tree and accession numbers in Genbank.

| Species | Abbreviation | Accession number |
| --- | --- | --- |
| *Aedes aegypti* | AaTFIIIB | XP_001655206.1 |
| *Apis florea* | AfTFIIIB | XP_003695250.1 |
| *Acyrthosiphon pisum* | ApTFIIB | XP_001951275.2 |
| *Arabidopsis thaliana* | AtBrf1 | NP_182035.2 |
| *Arabidopsis thaliana* | AtBrf2 | NP_187547.2 |
| *Arabidopsis thaliana* | AtBrf3 | NP_178237.3 |
| *Arabidopsis thaliana* | AtpBrp | CAC82714.1 |
| *Arabidopsis thaliana* | AtTFIIB | NP_181694.1 |
| *Brachypodium distachyon* | BdpBrp | XP_003558588.1 |
| *Chondrus crispus* | CcTFIIB | XP_005715543.1 |
| *Caenorhabditis elegans* | CeBrf1 | NP_495526.1 |
| *Cyanidioschyzon merolae* | CmpBrp | XP_005536374.1 |
| *Cyanidioschyzon merolae* | CmTFIIB | XP_005536732.1 |
| *Cyanidioschyzon merolae* | CmTFIIIB | XP_005535026.1 |
| *Chlorella variabilis* | CvBrf | EFN55262.1 |
| *Dictyostelium discoideum* | DdTFIIB | XP_635486.1 |
| *Drosophila melanogaster* | DmBrf | AAF72066.1 |
| *Guillardia theta* | GtpBrp | XP_005827035.1 |
| *Homo sapiens* | HsBrf1-1 | NP_001510.2 |
| *Homo sapiens* | HsBrf1-4 | NP_001229715.1 |
| *Homo sapiens* | HsBrf1-5 | NP_001229716.1 |
| *Homo sapiens* | HsBrf1-6 | NP_001229717.1 |
| *Homo sapiens* | HsBrf1-8 | NP_001229719.1 |
| *Hydra vulgaris* | HvBrf | XP_002161156.2 |
| *Mus musculus* | MmTFIIIB | NP_082469.2 |
| *Oryzias latipes* | OlTFIIIB | XP_004082772.1 |
| *Oryza sativa* | OsBrf | NP_001055147.1 |
| *Ostreococcus tauri* | OtTFIIIB | XP_003082641.1 |
| *Phytophthora sojae* | PhsoBrf | EGZ11170.1 |
| *Picea sitchensis* | PisiBrf | ABR17974.1 |
| *Physcomitrella patens* | PpBrf1 | XP_001770181.1 |
| *Prunus persica* | PppBrp | XP_007203626.1 |
| *Physcomitrella patens* | PpTFIIB | XP_001772159.1 |
| *Phaeodactylum tricornutum* | PtTFIIIB | XP_002185060.1 |
| *Saccharomyces cerevisiae* | ScBrf1 | NP_011762.1 |
| *Saccharomyces cerevisiae* | ScTFIIB | AAT93251.1 |
| *Selaginella moellendorffii* | SmBrf | XP_002981027.1 |
| *Selaginella moellendorffii* | SmpBrp | XP_002968085.1 |
| *Selaginella moellendorffii* | SmTFIIB | XP_002966817.1 |
| *Trichoplax adhaerens* | TaBrf | XP_002118016.1 |
| *Volvox carteri* | VcBfr1 | ADI46871.1 |
| *Vitis vinifera* | VvBrf1 | XP_002269372.1 |
| *Vitis vinifera* | VvBrf2 | XP_002269372.2 |
| *Vitis vinifera* | VvBrf3 | CBI31214.3 |
| *Vitis vinifera* | VvpBrp | XP_002267728.1 |
| *Vitis vinifera* | VvTFIIB | XP_002269045.1 |
| *Zea mays* | ZmBrf | NP_001141990.1 |
| *Zea mays* | ZmpBrp | AFW89355.1 |
